# Bird ticks in Hungary reflect western, southern, eastern flyway connections and two genetic lineages of *Ixodes frontalis* and *Haemaphysalis concinna*

**DOI:** 10.1186/s13071-016-1365-0

**Published:** 2016-02-24

**Authors:** S. Hornok, B. Flaisz, N. Takács, J. Kontschán, T. Csörgő, Á. Csipak, B. R. Jaksa, D. Kováts

**Affiliations:** Department of Parasitology and Zoology, Faculty of Veterinary Science, Szent István University, Budapest, Hungary; Plant Protection Institute, Centre for Agricultural Research, Hungarian Academy of Sciences, Budapest, Hungary; Department of Anatomy, Cell- and Developmental Biology, Eötvös Loránt University, Budapest, Hungary; Ócsa Bird Ringing Station, Ócsa, Hungary; Department of Evolutionary Zoology and Human Biology, University of Debrecen, Debrecen, Hungary

**Keywords:** *Ixodes*, *Haemaphysalis*, *Hyalomma*, Bird migration, COI gene, 16S rDNA gene

## Abstract

**Background:**

Birds play an important role in short- and long-distance transportation of ticks and tick-borne pathogens. The aim of the present study was to provide comprehensive information on the species and genetic diversity of ixodid ticks transported by migratory and non-migratory bird species in Central Europe, and to evaluate relevant data in a geographical, as well as in an ecological context.

**Methods:**

During a three year period (2012-2014), altogether 3339 ixodid ticks were collected from 1167 passerine birds (representatives of 47 species) at ringing stations in Hungary. These ticks were identified, and the tick-infestations of bird species were compared according to various traits. In addition, PCR and sequencing of part of the cytochrome oxidase subunit-I (COI) and 16S rDNA genes were performed from representatives of five tick species.

**Results:**

The most abundant tick species found were *Ixodes ricinus* and *Haemaphysalis concinna* (with 2296 and 989 immature stages, respectively). In addition, 48 *I. frontalis* (all stages), three *Hyalomma rufipes* nymphs, one *I. lividus* and two *I. festai* females were collected. The majority of *I. ricinus* and *I. frontalis* specimens occurred on ground-feeding bird species, as contrasted to *Ha. concinna. Hy. rufipes* showed the highest degree of sequence identity to an Ethiopian hybrid of the same tick species. Based on both COI and 16S rDNA gene analyses, two genetic lineages of *I. frontalis* were recognized (with only 91.4 % identity in their partial COI gene). These were highly similar to South-Western European isolates of the same tick species. Phylogenetic analysis of *Ha. concinna* specimens collected from birds in Hungary also revealed two genetic lineages, one of which showed high (≥99 %) degree of 16S rDNA sequence identity to conspecific East Asian isolates.

**Conclusions:**

Two genetic lineages of *I. frontalis* and *Ha. concinna* are transported by birds in Central Europe, which reflect a high degree of sequence identity to South-Western European and East Asian isolates of the same tick species, respectively. In addition, *I. festai* was collected for the first time in Hungary. These findings highlight the importance of western and eastern migratory connections by birds (in addition to the southern direction), which are also relevant to the epidemiology of tick-borne diseases.

## Background

The epidemiological role of birds has been increasingly recognized. They are carriers of important viruses, bacteria and parasites, some of which may pose a risk to humans and domestic or game/wild animals [1]. In Europe, similarly to other parts of the globe, migratory birds play an important role in short- and long-distance transportation of ixodid ticks (Acari: Ixodidae) and tick-borne pathogens [1]. Non-migrating bird species contribute significantly to the local tick populations, as they are preferred hosts of immature stages (larvae and nymphs) of several tick species of medical and veterinary importance, such as *Ixodes ricinus* [2] and *Haemaphysalis concinna* [3]. Since numerous exotic and local tick species are transported by birds and may infest humans, avian hosts may contribute to zoonotic pathogen transmission, particularly in urban habitats [4]. Furthermore, birds may harbour ornithophilic tick species, such as *I. frontalis* and *I. arboricola* [5], which may be relevant to the transmission of pathogens within or between bird populations [6].

Accordingly, most molecular studies focus on the detection of pathogens that are associated with bird ticks [1], or on avian hosts as potential pathogen reservoirs [7]. Reports on the molecular taxonomic comparison of birds ticks are rare (e.g. [5]), particularly in a geographical context [8].

In the eastern part of Central Europe (including Poland, Slovakia and Hungary) birds were reported to harbour exotic tick species [4, 9, 10]. It has also been shown that tick-carrier birds are important as hosts of local/indigenous tick species in the region [11, 12]. The large scale survey of this study aimed to extend the scope of these previous works by providing comprehensive information on the species and genetic diversity of ixodid ticks transported by migratory and non-migratory bird species in Central Europe, while evaluating relevant data in a geographical, as well as in an ecological context.

## Methods

### Sample collection

During a three year period (from January 2012 until December 2014) ixodid ticks were collected from passerine birds at three ringing stations in Hungary (Ócsa: 47.2967°, 19.2101°; Fenékpuszta: 46.7088°, 17.2427°; Bódva-völgy: 48.2934°, 20.7385°). Birds were captured by standard Ecotone mist-nets (Gdynia, Poland), 12 m in length, 2.5 m in height and with 16 mm mesh as described [7]. All captured birds were examined for the presence of ticks, which were removed with fine tweezers and put into 70 % ethanol (for storage) in separate vials according to their hosts. Morphological identification was done with a stereo microscope (SMZ-2 T, Nikon Instruments, Japan, illuminated with model 5000-1, Intralux, Switzerland) according to standard keys [13, 14]. Characteristics (feeding site preference, migration distance and weight) were assigned to bird species based on ornithological observations [15].

### Molecular analyses

The DNA was extracted from 46 specimens of *I. frontalis*, 12 larvae/nymphs of *Ha. concinna*, and one *Hyalomma* nymph, as well as from one hind leg of two *I. festai* and one *I. lividus* females as described [7].

The cytochrome oxidase subunit I (COI) gene was chosen as the first target for molecular analysis, on account of its suitability as a DNA-barcode sequence for tick species identification. The PCR was modified from Folmer et al. [16] and amplifies a 710 bp fragment of the gene. The primers HCO2198 (5′-TAA ACT TCA GGG TGA CCA AAA AAT CA-3′) and LCO1490 (5′-GGT CAA CAA ATC ATA AAG ATA TTG G-3′) were used in a reaction volume of 25 μl, containing 1 U (0.2 μl) HotStarTaq Plus DNA polymerase, 2.5 μl 10× CoralLoad Reaction buffer (including 15 mM MgCl_2_), 0.5 μl PCR nucleotide Mix (0.2 mM each), 0.5 μl (1 μM final concentration) of each primer, 15.8 μl ddH_2_O and 5 μl template DNA. For amplification, an initial denaturation step at 95 °C for 5 min was followed by 40 cycles of denaturation at 94 °C for 40 s, annealing at 48 °C for 1 min and extension at 72 °C for 1 min. Final extension was performed at 72 °C for 10 min.

Another PCR [17] was used to amplify a 460 bp fragment of the 16S rDNA gene from one sample among those that yielded the same COI genotype, with the primers 16S + 1 (5′-CTG CTC AAT GAT TTT TTA AAT TGC TGT GG-3′) and 16S-1 (5′-CCG GTC TGA ACT CAG ATC AAG T-3′). Other reaction components, as well as cycling conditions were the same as above, except for annealing at 51 °C.

PCR products were electrophoresed in a 1.5 % agarose gel (100 V, 60 min), stained with ethidium-bromide and visualized under ultra-violet light. Purification and sequencing was done by Biomi Inc. (Gödöllő, Hungary). Sequences were submitted to GenBank (Table 1). The phylogenetic analyses were conducted with the Tamura-Nei model and Maximum Composite Likelihood method by using MEGA version 5.2 as reported [18].

**Table 1:** Tick species, genotypes and GenBank accession numbers of sequences obtained in this study

Tick species	Accession number for part of the:	
	COI gene (corresponding genotypes)	16S rDNA gene (corresponding genotypes)
*Ixodes frontalis*	KU170492-500 (A-Hu1 to A-Hu9)	KU170518 (A-Hu16S)
	KU170501-9 (B-Hu1 to B-Hu9)	KU170519 (B-Hu16S)
*Ixodes festai*	-	KU170521-2 (Hu165, Hu166)
*Ixodes lividus*	KU170510	KU170520
*Hyalomma rufipes*	KU170491	KU170517
*Haemaphysalis concinna*	KU170511-6 (Hu1 to Hu6)	KU170523-5 (Hu167 to Hu169)

### Ethical approval

The study was carried out according to the national animal welfare regulations (28/1998). Licence for bird ringing was provided by the National Inspectorate for Environment and Nature (No 14/3858-9/2012.).

### Statistical analysis

Exact confidence intervals (CI) for the prevalence rates were calculated at a 95 % level. Sample prevalence data were analyzed by Fisher’s exact test. Mean values for the intensity of tick infestation (number of all ticks collected from a bird species, divided by the number of all tick-infested individuals of the same bird species) were compared between bird categories by Mann-Whitney *U*-Test. Differences were considered significant when *P* < 0.05.

## Results

### Tick-infestation of birds according to tick and bird species

In the period between 2012-2014, altogether 3339 ixodid ticks were collected from 1167 passerine birds (representatives of 47 species). The most abundant tick species found were *I. ricinus* and *Ha. concinna*, with 2296 (68.8 %, CI: 67.2–70.3 %) and 989 (29.6 %, CI: 28.1–31.2 %) specimens (only larvae and nymphs), respectively. The presence of *I. ricinus* on birds was noted between March and November, and that of *Ha. concinna* (both larvae and nymphs) from March to October.

**Fig. 1:**
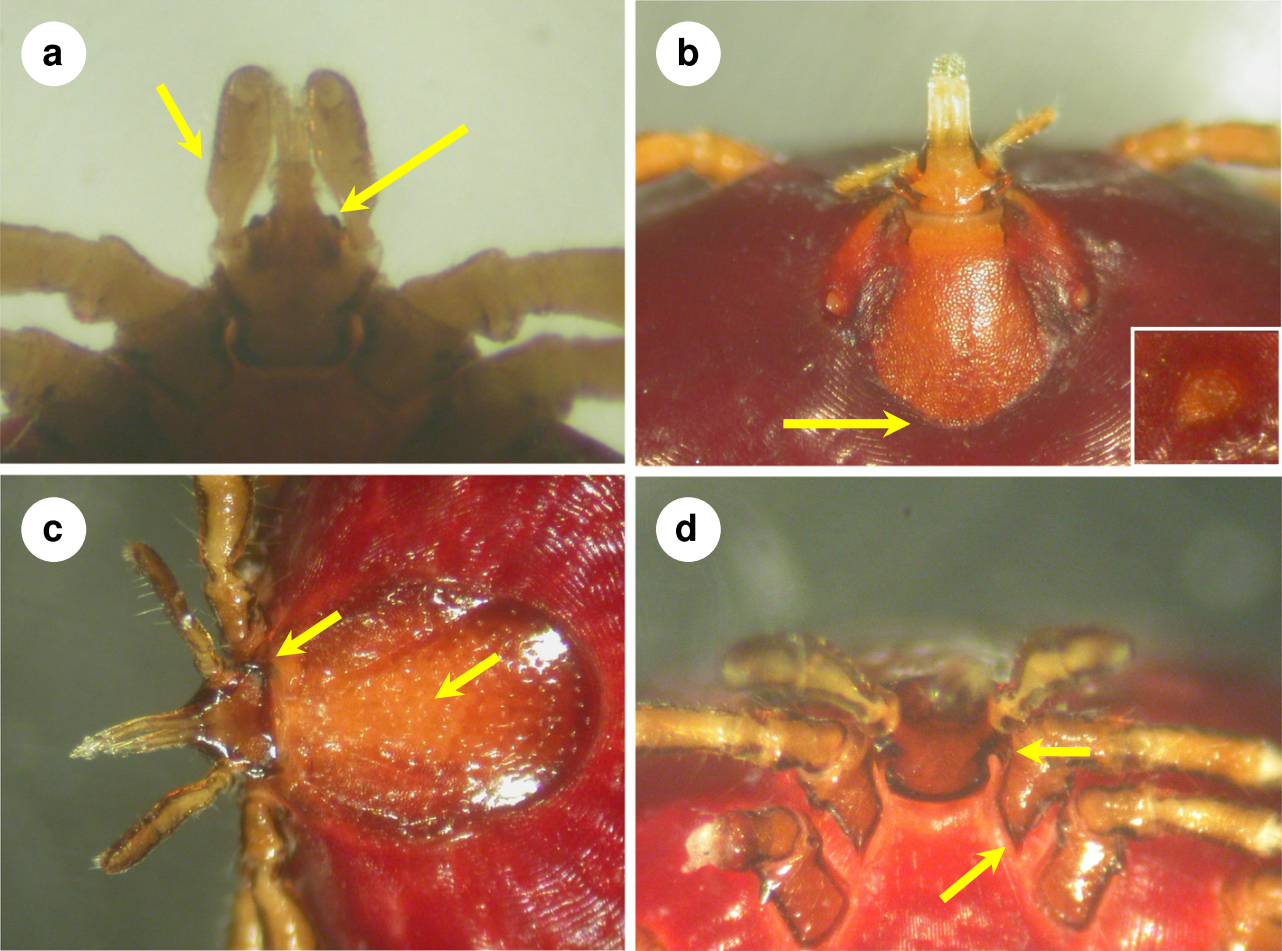
Morphology of tick species identified in the relevant stage for the first time in Hungary. **a**: *I. frontalis* nymph showing parallel sides of palps and “frons” (arrows); **b**: *Hy. rufipes* nymph with broadly rounded posterior margin of the scutum (arrow) and elongated spiracular plate (insert); **c**: *I. festai* female, dorsal view – the scutum with deep punctuations and few long hairs, distinct cornuae on the basis capituli (arrows); **d**: *I. festai* female, ventral view – broad auriculae curved backwards, long internal spur on coxa I (arrows)

Forty-eight *I. frontalis* specimens (including three adults) were also collected (Fig. 1a), with a majority (79.2 %, CI: 65–89.5 %) from Robins (*Erithacus rubecula*) (Table 2). This tick species occurred during all seasons (August to November and January to April), however, most specimens were collected in springtime (Table 3).

**Table 2:** Traits and tick-infestation of most important bird species in this study (of which at least eight tick-infested individuals were captured or at least 10 ticks were collected between March 2012 and November 2014)

Bird species characteristics					t/n	Cumulative number of tick specimens						
						*I. ricinus*		*H. concinna*		*I. fr.*	*I. fe.*	*H. r.*
Species^a^	Feeding	Migration	Weight (g)	n		L	N	L	N	L/N/F	F	N
ACR PAL	ARBOREAL	long	10–17	53	2.1	39	41	18	14	-	-	-
ACR SCH			10–13	30	2.6	3	10	10	56	-	-	-
ACR SCI			9–12	70	1.8	29	45	18	36	-	-	-
LOC LUS			14–17	92	4.9	1	7	206	236	-	-	-
LOC NAE			13–16	2	11	-	-	18	4	-	-	-
PHY COL		middle	6–11	8	1.4	5	6	-	-	-	-	-
SYL ATR			16–25	69	1.7	45	39	7	22	-/1/-	-	-
CAR CHL		short	25–35	30	1.7	1	47	-	1	-/-/1	1	-
COC COC			**46–80**	11	2.4	-	25	1	1	-	-	-
EMB CIT		local	27–30	2	20	-	-	38	2	-	-	-
EMB SCH			27–30	2	8.5	3	-	2	12	-	-	-
PAR MAJ			16–22	81	1.8	49	88	2	1	1/-/1	-	-
LUS LUS	GROUND	long	24–38	10	4.2	40	2	-	-	-	-	-
LUS MEG			17–28	24	4	61	17	18	1	-	-	-
SYL COM			13–20	12	1.1	5	8	-	-	-	-	3
TUR ILI			**55–75**	3	6.5	2	12	-	-	-	-	-
ERI RUB		short	16–22	318	2.3	469	195	27	5	22/15/1	-	-
PRU MOD			16–25	67	3.1	24	173	3	4	-	1	-
TRO TRO			7–12	13	1.8	15	8	-	-	-	-	-
TUR MER			**80–140**	149	4.6	137	421	47	73	-	-	-
TUR PHI			**65–95**	56	4.5	49	118	31	50	5/1/-	-	-

Bold numbers indicate weight of bird species in the larger body weight category. The cumulative number of tick specimen refers to the number of larvae, nymphs or female ticks collected from all individuals of the relevant bird species during the study period

Abbreviations: *n* number of tick-infested individuals, *t/n* mean intensity of tick infestation (number of all ticks divided by the number of all tick-infested birds), *L* larva, *N* nymph, *F* female, *I. fr.* - *Ixodes frontalis*; *I. fe.* - *Ixodes festai*; *H. r.* - *Hyalomma rufipes*

^a^ACR = *Acrocephalus palustris*, ACR SCH = *A. schoenobaenus*, ACR SCI = *A. scirpaceus*, LOC LUS = *Locustella luscinioides*, LOC NAE = *L. naevia*, PHY COL = *Phylloscopus collibita*, SYL ATR = *Sylvia atricapilla*, CAR CHL = *Carduelis chloris*, COC COC = *Coccothraustes coccothraustes*, EMB CIT = *Emberiza citrinella*, EMB SCH = *E. schoeniclus*, PAR MAJ = *Parus major*, LUS LUS = *Luscinia luscinia*, LUS MEG = *L. megarhynchos*, SYL COM = *S. communis*, TUR ILI = *Turdus iliacus*, ERI RUB = *Erithacus rubecula*, PRU MOD = *Prunella modularis*, TRO TRO = *Troglodytes troglodytes*, TUR MER = *T. merula*, TUR PHI = *T. philomelos*

**Table 3:** Genotypes of *Ixodes frontalis* and *Haemaphysalis concinna* identified in this study, according to bird species and season

	Genotype		Bird species							
	COI	16S rDNA	ERI RUB			TUR PHI	PAR MAJ	SYL ATR	CAR CHL	
*I. frontalis*	A-Hu1	A-Hu16S	S^1^ S^1^ S^1^ S^2^ S^2^ S^2^ S^8^ S S S S S A			S^5^	S			
	A-Hu2		S^2^							
	A-Hu3		S							
	A-Hu4		S S							
	A-Hu5		S^4^ S^4^							
	A-Hu6		S							
	A-Hu7								w	
	A-Hu8					A^7^				
	A-Hu9		S^8^							
	B-Hu1	B-Hu16S	S^1^ S^1^ S S			M				
	B-Hu2		S^1^							
	B-Hu3		S^2^ S S S				A			
	B-Hu4							S		
	B-Hu5		S							
	B-Hu6					S^5^				
	B-Hu7		S^6^ S^6^ S^6^ S^6^							
	B-Hu8					A^7^ A^7^				
	B-Hu9		A							
	COI	16S rDNA	ERI RUB	ACR SCH	TUR MER	PRU MOD	SYL NIS	SYL ATR	LOC LUS	EMB CIT
*H. concinna*	Hc-Hu1	Hu167		M						
	Hc-Hu2						M		M	
	Hc-Hu3				MA			S		M
	Hc-Hu4	Hu168			S					
	Hc-Hu5		S A			S				
	Hc-Hu6	Hu169	A							

The number of letters of a season below one bird species in the given row indicates the number of ticks belonging to the relevant genotype. The same upper index on these letters indicate ticks that were found simultaneously on the same bird individual

*Abbreviations*: *S* spring, *M* summer, *A* autumn, *W* winter. For abbreviations of bird names see Table 1

Regarding exotic tick species, three *Hyalomma* nymphs, which resembled *Hy. rufipes* based on the spiracular plates and the scutum (Fig. 1b), were found on a Common Whitethroat (*Sylvia communis*) in May (2014).

In addition, two *I. festai* females, identified on morphological bases (Fig. 1c, d), were removed from a Greenfinch (*Carduelis chloris*) and a Dunnock (*Prunella modularis*) in March (2014). One *I. lividus* female was collected from a Sand Martin (*Riparia riparia*) in July (2014).

Among the most important tick-infested bird species in this survey (Table 2), the majority of *I. ricinus* and *I. frontalis* larvae/nymphs (1756 of 2239: 78.4 %, CI: 76.7–80.1 % and 44 of 48: 91.7 %, CI: 80–97.7 %, respectively) occurred on ground-feeding bird species, whereas 73.1 % of *Ha. concinna* immatures (705 of 964, CI: 70.2–75.9 %) were found on arboreal birds, reflecting a highly significant difference (Fisher’s exact test: *P* < 0.0001). On the other hand, the intensity of tick infestation (Table 2) had no significant association with bird species of smaller (6–30 g) or larger (31–140 g) body weight, or with bird species that have long vs. short distance (or no) migration (Mann-Whitney *U*-Test: *P* > 0.05).

### Molecular taxonomic analysis of bird ticks in a geographical context

#### Ixodes frontalis

**Fig. 2:**
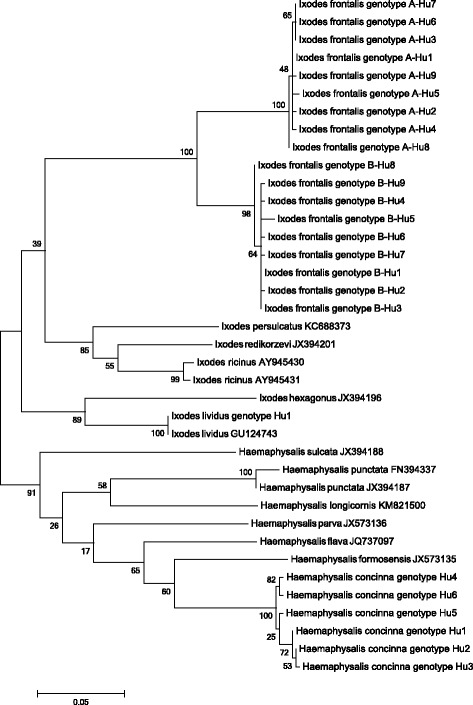
Phylogenetic relationships of *Ixodes* and *Haemaphysalis* sp. ticks based on COI gene. Specimens collected in this study (genotypes with “Hu”) and related data from GenBank are included. Branch lengths correlate to the number of substitutions inferred according to the scale shown

**Fig. 3:**
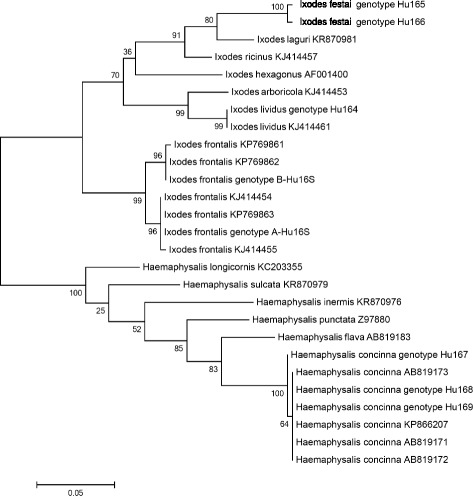
Phylogenetic comparison of 16S rDNA sequences of *Ixodes* and *Haemaphysalis* sp. ticks. Specimens identified in the present study (genotypes including Hu) and other sequences from GenBank are included. Branch lengths correlate to the number of substitutions inferred according to the scale shown

Among the 46 *I. frontalis* specimens for which part of the COI gene was sequenced, two genetic lineages (each containing nine genotypes) were clearly recognizable (“A”: KU170492-500, and “B”: KU170501-9) and the separation of these lineages had high bootstrap support on the phylogenetic tree (Fig. 2). The relevant genotypes had 1-2 nucleotide differences within lineage “A” and 1-4 within lineage “B”, but up to 56 nucleotide difference (598/654 bp, i.e. only 91.4 % identity) between the two lineages. The subsequent 16S rDNA gene analysis included DNA samples of each different COI genotype, but revealed only two distinct genetic variants (KU170518: genotype A-Hu16S, KU170519: genotype B-Hu16S), which showed a 4 bp difference, i.e. 99 % (402/406 bp) identity. These two 16S rDNA genotypes had 100 % sequence identity to South-Western European isolates (KP769863 and KP769862, respectively, from Azores). The phylogenetic analyses of 16S rDNA sequences confirmed the separation of two *I. frontalis* lineages (Fig. 3). The isolation sources of *I. frontalis* genotypes are shown in Table 3.

#### Ixodes festai

In the case of *I. festai* the sequencing of the amplified part of the COI gene was not successful. The 16S rDNA sequences of the two specimens (KU170521-2) differed in three nucleotides (373/376 bp, i.e. 99.2 % identity), but clustered together on the phylogenetic tree (Fig. 3).

#### Ixodes lividus

The COI sequence of *I. lividus* obtained in this study (KU170510) had 100 % identity with an isolate of the same tick species from the UK (GU124743). The partial 16S rDNA sequence of the Hungarian specimen (KU170520) had 99.7 % (398/399 bp) identity with another isolate from Western Europe, Belgium.

#### Hyalomma rufipes

The partial COI sequence of one *Hyalomma* nymph (KU170491) showed the highest (645/649 bp, i.e. 99.4 %) degree of identity to a *Hy. rufipes* × *Hy. dromedarii* hybrid from Ethiopia (AJ437079), whereas a 99.2 % (644/649 bp) identity to *Hy. marginatum* (AJ437091). Based on the partial sequence of its 16S rDNA gene (KU170517), this specimen showed the highest degree of identity to *Hy. rufipes* (405/406 bp, i.e. 99.8 % identity to L34307, and only 403/407 bp: 99 % identity to KP776645, *Hy. marginatum*).

#### Haemaphysalis concinna

Among the molecularly analysed 12 *Ha. concinna* specimens six different COI genotypes were found (KU170511-6), with a difference in up to eight nucleotides (meaning 622/630, i.e. 98.7 % identity). The six COI genotypes clustered in two lineages (supported by high bootstrap value) on the phylogenetic tree (Fig. 2). These COI genotypes represented three 16S rDNA genotypes (KU170523-5), with 1–2 bp differences. The 16S rDNA phylogenetic analysis confirmed the separation of genotype Hu167 (encompassing COI genotypes Hu1-3) from the others (Hu168-9) (Fig. 3). The latter (e.g. KU170524) showed high (99.7 %, i.e. 387/388 bp) degree of identity to Far Eastern isolates of *Ha. concinna* (from Japan: e.g. AB819171) and another from East Siberia (KP866207: 384/387 bp, 99.2 % identity), with which they clustered together on the phylogenetic tree (Fig. 3). The isolation sources (bird species) and seasonality of COI and 16S rDNA genotypes of *Ha. concinna* are shown in Table 3.

## Discussion

In the present study molecular evidence is provided for the first time on the transportation of immature stages of *Hy. rufipes* by birds in Central Europe. In addition, *I. festai* is reported for the first time in Hungary. While two females of *I. frontalis* (infesting birds) had been reported in Hungary more than half a century ago [19], the present results attest that this tick species is transported by birds regularly in the region. Interestingly, another ornithophilic tick species, *I. arboricola* was not found in this survey, although its preferred hosts (i.e. cavity-nesting bird species) were included in the study, and it has formerly been reported in Hungary [13] as well as in neighbouring countries (Slovakia: [6]; Romania: [20]).

The seasonal presence of *I. ricinus* on birds coincided with the reported questing activity of this tick species in Hungary [21]. In contrast to this, *Ha. concinna* larvae or nymphs were found two or one months earlier (respectively) on avian hosts (i.e. from March), compared to the initiation of their known seasonal activity in the region [3].

The presence of *I. ricinus* and *I. frontalis* larvae/nymphs showed a significant association with ground-feeding bird species (demonstrated here for the first time in case of *I. frontalis*), whereas *Ha. concinna* larvae and nymphs occurred significantly more frequently on arboreal birds. The latter finding can be explained by the relatively high questing height of *Ha. concinna* larvae and nymphs on the vegetation, as an adaptation to the size of their preferred host species (roe deer) in the region [22].

In the present study the great majority of *I. frontalis* specimens were collected from Robins. This bird species is known to have predominantly south-west to north-east spring migration from the Mediterranean region to Hungary [23]. Therefore, the present data suggest that *I. frontalis* is mainly transported from South-Western Europe to Central Europe. In support of this migratory connection, the 16S rDNA genotypes reported here were 100 % identical to corresponding sequences from the Azores, and the spring predominance of *I. frontalis* larvae and nymphs on birds in Hungary (as observed in the present study) followed the late winter seasonal peak reported in Portugal [24].

Based on the analysis of two genetic markers, the present data clearly indicate that two distinct genetic lineages of *I. frontalis* are transported by birds in Central Europe. The separation of these lineages is supported by high bootstrap values on the COI and 16S rDNA phylogenetic trees (Figs. 2, 3). Interestingly, the degree of COI sequence divergence between the two lineages (9 %) exceeds the proposed approximated sequence difference as species boundary for ticks (6.1 % of COI gene: [25]).

Previously, *I. festai* was not reported from Hungary. In the present study this tick species was found on a Greenfinch and a Dunnock. Both bird species migrate for wintering to the Mediterranean Basin, in the direction of Italy [15], where *I. festai* is known to occur [26].

The tick *I. lividus* is host specific for the Sand Martin (*Riparia riparia*). The sequence identity between the isolates of this tick species from Hungary (reported here) and the UK [27] indicates that the same genotype is present in Western and Central European populations of *I. lividus*, despite the fact that no direct migration of Sand Martins is known between Hungary and the UK [15].

In the present study *Hyalomma* nymphs were removed from a Common Whitethroat. Populations of this bird species, which breed in Central Europe, are known to migrate to sub-Saharan Africa for wintering [15]. Morphologically all three ticks resembled *Hy. rufipes*, and one of them showed the closest identity in its partial COI gene to an Ethiopian *Hy. rufipes* hybrid. The GenBank reference strains used for comparison in this context were reliably identified according to taxonomic keys [28]. In general, larvae and nymphs of the *Hy. marginatum* complex are known to be transported by birds to both Western and Central Europe from the south [10, 29]. *Hy. rufipes* in particular, was reported on birds in Northern Europe [30]. In Europe, birds carrying *Hy. rufipes* most likely arrive from the Middle East (which is a stopover region along the Black Sea – Mediterranean flyway), where larvae and nymphs of this tick species predominate on birds [31, 32]. Importantly, even under the continental climate in Central Europe nymphs of *Hy. rufipes* are able to moult to adults, previously reported to infest cattle in Hungary [33].

Based on the present results *Ha. concinna* larvae/nymphs carried by song birds in Central Europe have a high degree of 16S rDNA gene identity with conspecific ticks from East Siberia [34] and the Far East, Japan [35]. Although the reasons for this close identity can be multiple, tick exchange between Central Europe and East Asia via migratory birds may significantly contribute to it. The East Atlantic and the Black Sea – Mediterranean flyways connect Europe to Asia (the latter being more relevant to the study area). Migratory birds wintering in Western Europe and those having spring migration towards Russian Far East may have overlapping breeding grounds [36]. In this survey *Ha. concinna* genotypes, highly similar to East Asian isolates, were only collected during spring and autumn bird migration, i.e. from Robins, a Blackbird (*Turdus merula*) and a Dunnock (Table 2). These bird species have eastern migratory connections, i.e. towards Eastern Europe and Asia [15, 37].

## Conclusions

Two genetic lineages of *I. frontalis* and *Ha. concinna* are transported by birds in Central Europe, which reflect a high degree of sequence identity to South-Western European and East Asian isolates of the same tick species, respectively. In addition, *I. festai* was collected for the first time in Hungary. These findings highlight the importance of western and eastern migratory connections by birds (in addition to the southern direction), which are also relevant to the epidemiology of tick-borne diseases.
